# Dietary acid load, depression, and anxiety: results of a population-based study

**DOI:** 10.1186/s12888-023-05163-3

**Published:** 2023-09-18

**Authors:** Hossein Bahari, Najmeh Seifi, Elahe Foroumandi, Fatemeh Kourepaz, Hediye Erfaniyan Shahabi, Kimia Ervin, Nahid Khabari, Gordon A. Ferns, Majid Ghayour-Mobarhan

**Affiliations:** 1 Student Research Committee, Mashhad University of Medical Sciences, Mashhad, Iran; 2 Department of Nutrition, Faculty of Medicine, Mashhad University of Medical Sciences, Mashhad, Iran; 3 International UNESCO Center for Health-Related Basic Sciences and Human Nutrition, Mashhad University of Medical Sciences, Mashhad, Iran; 4 Non-Communicable Diseases Research Center, Department of Nutrition and Biochemistry, Faculty of Medicine, Sabzevar University of Medical Sciences, Sabzevar, Iran; 5 Varastegan Institute for Medical Sciences, Mashhad, Iran; 6 Department of Biology, Faculty of Sciences, Mashhad Branch of Islamic Azad University, Mashhad, Iran; 7 Division of Medical Education, Brighton and Sussex Medical School, Falmer, Brighton, Sussex UK; 8 Metabolic Syndrome Research Center, Mashhad University of Medical Sciences, Mashhad, 99199-91766 Iran

**Keywords:** Dietary acid load, Potential renal acid load, Depression, Anxiety

## Abstract

**Background:**

Dietary acid load seems to be associated with psychological disorders through several mechanisms, and may barricade their treatment and recovery. The aim of current study was to evaluate the relationship between dietary acid load, using potential renal acid load (PRAL) and dietary acid load (DAL) scores, with the severity of depression and anxiety among Iranian adults.

**Methods:**

A total of 6531 participants aged 35–65 years were recruited in this cross-sectional study. A validated food frequency questionnaire (FFQ) was used to assess dietary intakes of participants. DAL was estimated using PRAL and DAL scores. Depression and anxiety were screened using Beck Depression Inventory (BDI) and Beck Anxiety Inventory (BAI).

**Results:**

In the fully adjusted model, women with the highest DAL or PRAL had higher odds of more severe depression compared to those in the lowest category (OR = 1.20; 95% CI = 1.03–1.42 and OR = 1.20; 95% CI = 1.03–1.39, respectively). No significant association was observed between DAL and PRAL and depression severity in men and in the population as a whole. Regarding the association between PRAL and anxiety severity, there was no significant association when separated by sex. However, in the fully adjusted model for the whole population, participants in the highest tertile of PRAL had 13% greater odds of more severe anxiety than the lowest tertile (OR: 1.13, 95% CI: 1.01–1.13). No significant association was observed between DAL and anxiety severity in women, men or in the whole population.

**Conclusion:**

Women, but not men, with higher dietary acid load had significantly higher odds of having more severe depression. A significant positive association was also observed between dietary acid load and anxiety in the whole population.

## Introduction

Depression and anxiety are the most prevalent mental health problems in most populations [1]. The world prevalence of depression and anxiety tends to be 31.4% and 31.9%, respectively [2]. According to the World Health Organization (WHO), poor mental health accounts for global economic losses of US$ 1 trillion per year [3]. The prevalence of mental disorders among the Iranian population aged 18–64 years over the past 12 months in 2011 was 23.6%, followed by 15.6% anxiety disorders [4]. Recent findings showed an increase in the prevalence of psychiatric disorders between 1999 and 2015 in Iranian adults [5]. Also, another investigation indicated that the prevalence of depression among Iranian patients was continuously growing from 2010 to 2018 and was estimated to be 34.26% (95% CI, 24.12 − 44.10%) [6].

The state of mental health affects people’s eating habits, as it has been shown that people suffering from depression and anxiety tend to consume more fat and sugar to compensate for their negative mental states [7, 8]. Nutritional psychiatry is an emerging discipline focused on the relationship between dietary patterns and mental health [9]. It has been hypothesized that healthy eating patterns from dietary recommendations and nutrient requirements may contribute to better mental health [10]. One of the main components of any diet is its content of foods that can affect acid–base balance [11]. Generally, consuming more animal products, phosphorus, and protein, such as meat, egg, and cheese, induces an acid load. Inversely, high amounts of vegetables and fruits high in potassium, magnesium, and calcium cause alkalis [12, 13].

The dietary acid load (DAL) is another index frequently used to evaluate diet quality in various populations. Different studies have estimated DAL based on dietary data and calculated the Potential renal net acid load (PRAL) and net endogenous acid production (NEAP). Higher and positive values of DAL and PRAL account for acid-forming potential [14]. Most recent studies have used PRAL and DAL to estimate diet-induced acid load [15–17].

Epidemiological studies have suggested that increasing acid load in the body by consuming acidogenic foods is associated with metabolic abnormalities, including diabetes, hypertension, osteoporosis, sarcopenia, and cardiovascular disorders [11]. Furthermore, two recent studies on American and Japanese populations have reported the association of high dietary acid load with a higher mortality rate [18, 19]. Some recent reports have also investigated the effect of acid-base balance on mental health. Daneshzad et al. reported a positive association between dietary acid load (high meat intake) and anxiety and depression [20]. At the same time, it has shown a protective role of a plant-based diet against these mental problems among diabetic women [20]. The same results were reported by another study among children [21]. Mozaffari et al. have shown a strong positive relationship between dietary acid-base load with depression, anxiety, and psychological distress among Iranian women [22]. Further, a recent study conducted on healthy adults showed a significant association between dietary acid load and depression and anxiety. This study estimated the dietary acid load using protein to potassium ratio (Pro: K) [23].

To the best of our knowledge, there are few community-based studies that have focused on the association of diet-induced acid load with depression and anxiety among adults. Therefore, the current cross-sectional study aimed to evaluate the relationship between dietary acid load, using PRAL and DAL scores, with depression and anxiety scores among the participants of the MASHAD cohort study in Iran.

## Methods

### Study design

This cross-sectional study was conducted on participants who were recruited as part of Mashhad Stroke and Heart Sclerotic Disorders (MASHAD) study [24]. A total of 6531 participants aged 35–65 years were recruited. Demographic data, anthropometric measurements, physical activity level (PAL), dietary intake, and depression and anxiety scores were determined in all patients. All experiments were performed following the declaration of Helsinki and Mashhad University of Medical Sciences ethical guidelines and regulations. The research protocol was approved by the School of Medicine, Mashhad University of Medical Sciences, and Biomedical Research Ethics Committee (IR.MUMS.MEDICAL.REC.1398.228). All participants signed a written informed consent before participating in the study.

### Anthropometric measurements

In all patients, body weight was measured, in light clothing and without shoes, using a calibrated counterweight balance (Seca, Japan). Height was also measured using a telescopic stadiometer, while the participant’s head was in the Frankfurt plane. Weight and height counts were rounded to the nearest 0.1 Kg and 0.1 cm, respectively.

Body mass index (BMI) was calculated as weight in kilograms divided by the square of height in meters.

### Physical activity level

Physical activity level (PAL) was defined using the James and Schofield human energy requirements equations. Estimation of energy requirement can be assessed as three components: basal metabolic rate (BMR), thermic response to food ingestion (TR), and PAL. The TR can be incorporated into PAL, estimate to provide two components, and the PAL may then be expressed as a multiple of BMR [25]. The physical activity questions based on James and Schofield equation were selected from the Scottish Heart health Study (SHHS)/ MONICA questionnaire [26]. Questions were divided into time spent on activities during work, non-work time, and in bed. Integrated energy index (IEI) for men at inactive, moderate, and active levels were 1.51, 2.49, and 4.34, respectively. IEI for women at inactive, moderate, and active levels were 1.61, 2.52, and 4.39, respectively. Considering time spent on each activity and the IEI, the total energy expenditure (TEE) was calculated. BMR was estimated using the FAO/WHO/UNU Eq. (28). PAL was calculated as TEE /BMR [29].Individuals with 1-1.39 physical activity levels were classified as inactive groups, and those with PAL between 1.4 and 1.59 and 1.6–1.89 were categorized as low activity and moderate activity groups, respectively. The physical activity level of high-activity subjects was 1.9–2.5 [25].

### Assessment of depression and anxiety

The Beck Depression Inventory II(BDI- II) [28] consists of 21 items assessing the severity of depression symptoms using statements scored from 0 to 3. The validated Persian version was used for this study (concurrent validity of 0.77, and reliability of 0.74) [29]. The following criteria were presented: normal (score of 0–10), mild mood disturbances (score of 11–16), and Clinical depression (moderate or severe, score of 17–63) [30].

The validated Persian version of Beck Anxiety Inventory (BAI) was used for assessing the symptoms of anxiety (validity of 0.83, and reliability of 0.72) [31, 32]. The BAI questionnaire contains 21 items, and each item achieves a score between 0 and 3. Accordingly, the final score can be between 0 (minimum) and 63 (maximum). Cut off scores are as follows: 0–7 = minimal or no anxiety, 8–15 = mild anxiety, 16–25 = moderate anxiety and 26–63 = severe anxiety [33].

### Dietary intakes

Dietary intakes were assessed using a validated 65-item food frequency questionnaire (FFQ) in the first phase of MASHAD study [34]. Experienced researchers administered the questionnaire in a face-to-face interview. Diet Plan 6 software (Forestfield Software Ltd., Horsham, West Sussex, UK) was used to analyze the energy and nutrient intakes. Food groups, including dairy, fruit and vegetables, meats, and grains, were constructed as follows: dairy: milk, yogurt, cheese, yogurt drink, cream, and ice-cream; fruits and vegetables: starch vegetables, green leafy vegetables, other vegetables, fresh fruits, and dried fruits; meats: red meat and processed meat, poultry meat, fish, and eggs; grains: whole-grain bread, white bread, rice, pasta, and biscuits.

### Dietary acid load

We used two scores to estimate the dietary acid load of each individual. The potential renal acid load (PRAL) and dietary acid load (DAL). The following formulas were used for calculations [35, 36]:

PRAL (mEq/d) = 0.4888× protein intake (g/d) + 0.0366×phosphorus (mg/d) – 0.0205× potassium (mg/d) – 0.0125× calcium (mg/d) – 0.0263 × magnesium (mg/d).

DAL (mEq/day) = PRAL+ (body surface area[m²] ×41[mEq/day]/1.73 m²)

Body surface area (m²) was calculated as [37, 38]: 0.007184 × height (cm) ^0.725^ × weight (kg) ^0.425^.

### Statistical analysis

We classified participants based on tertile cut-off points of DAL score. General characteristics of study participants across tertiles of DAL score were presented as means ± SDs for continuous variables and frequency number (percentages) for categorical variables. To examine the differences across tertiles, we used ANOVA for continuous variables and chi-square test for categorical variables. We also used multivariable ordinal logistic regression to estimate ORs and 95% CIs for assessing depression (normal/ mild mood disturbances/ clinical depression) and anxiety (normal/mile/ moderate/ severe) severity across tertiles of DAL and PRAL scores in crude and multivariable-adjusted model. In the first model, we adjusted for age (continuous) and energy intake (continuous). Additionally, adjustments were made for marital status (married/ single), education level (under university/university educated), smoking status (current/ ex-/ non-smoker), physical activity level (continuous), chronic diseases including diabetes, hypertension, or dyslipidemia (yes/no). Diabetes was defined as fasting blood glucose above 126 mg/dl or history of diabetes. Hypertension was defined as systolic blood pressure above 140 mmHg, or diastolic blood pressure above 90 mmHg, or history of hypertension. Dyslipidemia was defined as total cholesterol greated than 200 mg/dl, or LDL-C above 130 mg/dl, or triglycerides above 150 mg/dl, or HDL-C below 40 mg/dl. In the full adjustment model, we further adjusted for BMI, dietary intake of fiber and mono-saturated fatty acids (MUFA). To obtain the overall trend of ORs across tertiles of PRAL and DAL scores, we considered these tertiles as ordinal variables. All statistical analyses were done using the Statistical Package for Social Sciences (version 25; SPSS Inc.). *P* < 0.05 was considered statistically significant.

## Results

A total of 6531 subjects were included in the final analysis. Overall, 33% (n = 2157) of study participants suffered from depression or were at the borderline level, and 52.7% (n = 3443) of them had some degree of anxiety. Baseline characteristics of participants by categories of DAL are provided in Table 1. Participants with a higher DAL were younger (47.86 vs. 48.93, p < 0.001), had a higher BMI (28.70 vs. 27.46, p < 0.05) and lower PAL (1.52 vs. 1.68, p < 0.001) than those with lower a DAL. There was significant difference in gender distribution, marital and smoking status across tertiles of DAL (p < 0.001). No significant difference was seen in the distribution of participants in terms of education level, presence of chronic diseases, and the prevalence of depression and anxiety categories across tertiles of DAL.

**Table 1:** Baseline characteristic of study participants by categories of DAL

	T1 (n = 2177)	T2 (n = 2177)	T3 (n = 2177)	p-value	p-trend
PRAL	-18.86 ± 11.13^*^	-4.10 ± 4.70	7.23 ± 8.26	< 0.001	< 0.001
DAL	21.17 ± 10.49	36.94 ± 2.99	50.41 ± 8.01	< 0.001	< 0.001
Age (year)	48.93 ± 8.26	48.30 ± 8.29	47.86 ± 8.07	< 0.001	< 0.001
Gender				< 0.001	-
Male, n (%)	1527 (70.14)^#^	1367 (62.79)	1045 (48)		
BMI (kg/m^2^)	27.46 ± 4.65	27.81 ± 4.67	28.70 ± 4.77	< 0.001	0.026
Marital status				< 0.001	-
Married, n (%)	2012 (92.42)	2008 (92.24)	2053 (94.30)		
Education level, n (%)				0.808	-
University graduated	234 (10.75)	225 (10.33)	238 (10.93)		
Smoking status, n (%)				< 0.001	-
Current smoker Ex-smoker	418 (19.2) 196 (9)	438 (20.12) 190 (8.73)	509 (23.38) 259 (11.90)		
PAL	1.68 ± 0.28	1.61 ± 0.28	1.52 ± 0.29	< 0.001	< 0.001
Chronic disease				0.270	
Yes, n (%)	1941 (89.16)	1957 (89.89)	1970 (90.49)		
Depression, n (%)				0.436	-
Normal	943 (43.32)	923 (42.50)	970 (44.60)		
Mild mood disturbances	518 (23.79)	502 (23.10)	513 (23.60)		
Clinical depression	716 (32.89)	748 (34.40)	690 (31.80)		
Anxiety score, n (%)				0.206	-
Normal	1019 (46.80)	1000 (46.0)	1068 (49.1)		
Mild anxiety	584 (26.80)	631 (29.0)	570 (26.2)		
Moderate anxiety	358 (16.40)	328 (15.1)	318 (14.6)		
Severe anxiety	216 (9.90)	214 (9.8)	219 (10.1)		

BMI: body mass index, DAL: dietary acid load, PAL: physical activity level, PRAL: potential renal acid load. ^*^ Continuous variables are presented as mean ± SD, ^#^ categorical variables are presented as number (percent). We used ANOVA for continuous variables and chi-square test for categorical variables

The dietary intakes of participants across tertiles of DAL are shown in Table 2. High DAL was associated with higher intakes of energy, carbohydrates, protein, fat, phosphorus, magnesium, grains and meats, as well as lower intakes of fruits, vegetables, dairy, fiber, total sugar, potassium and calcium (P < 0.001 for all). There was no significant trend association between magnesium intake and tertiles of DAL.

**Table 2:** Nutritional intakes of study participants by categories of DAL

	T1 (n = 2177)	T2 (n = 2177)	T3 (n = 2177)	p-value	p-trend
Food groups					
Grains (g/day)	304.73 ± 129.94	344.92 ± 138.77	428.22 ± 176.13	< 0.001	< 0.001
Fruits(g/day)	346.75 ± 264.14	199.70 ± 147.61	157.27 ± 123.07	< 0.001	< 0.001
Vegetables(g/day)	362.42 ± 169.90	251.63 ± 107.27	216.08 ± 102.39	< 0.001	< 0.001
Meats(g/day)	97.07 ± 43.01	99.73 ± 42.66	131.37 ± 72.51	< 0.001	< 0.001
Dairy(g/day)	382.71 ± 233.75	345.22 ± 217.60	345.05 ± 201.89	< 0.001	< 0.001
Energy and nutrients					
Energy (Kcal/day)	1936.44 ± 576.37	1871.33 ± 527.31	2165.22 ± 616.95	< 0.001	< 0.001
Carbohydrates (g/day)	281.51 ± 92.96	268.67 ± 83.97	300.43 ± 91.88	< 0.001	< 0.001
Protein (g/day)	71.20 ± 20.42	70.30 ± 19.31	84.40 ± 25.13	< 0.001	< 0.001
Fat (g/day)	63.69 ± 24.52	62.28 ± 23.11	73.76 ± 31.16	< 0.001	< 0.001
Fiber (g/day)	27.43 ± 9.85	24.00 ± 8.92	26.33 ± 10.45	< 0.001	< 0.001
Total sugar (g/day)	146.70 ± 59.07	123.09 ± 46.37	122.89 ± 48.06	< 0.001	< 0.001
Potassium (mg/day)	3837.78 ± 1102.27	3074.52 ± 831.75	3090.88 ± 869.99	< 0.001	< 0.001
Magnesium (mg/day)	332.19 ± 99.43	302.34 ± 93.01	337.34 ± 109.09	< 0.001	0.092
Calcium (mg/day)	995.51 ± 346.66	885.30 ± 232.72	960.81 ± 329.40	< 0.001	< 0.001
Phosphorus (mg/day)	1294.56 ± 383.05	1226.84 ± 357.48	1406.67 ± 402.00	< 0.001	< 0.001

Variables are presented as mean ± SD. We used ANOVA to compare means

The multivariable-adjusted odds ratios (ORs) and 95% confidence interval (CIs) for depression severity across tertiles of DAL and PRAL are indicated in Table 3. Female participants with the highest DAL had a higher odds of more severe depression compared to those in the lowest category (OR = 1.20; 95% CI = 1.03–1.39). After adjusting for age and energy intake in model 1, the association remained significant in females (OR: 1.24; 95% CI: 1.07–1.44). In the fully adjusted model, females with the highest DAL had 21% higher odds of more severe depression compared to those in the lowest category (OR = 1.21; 95% CI = 1.03–1.42).

Regarding PRAL tertiles, females with the highest PRAL had a higher odds of more severe depression compared to those in the lowest category in model 1 and model 3(OR = 1.15; 95% CI = 1.00–1.33 and OR = 1.17; 95% CI = 1.00–1.36, respectively). No significant association was observed between DAL and PRAL and depression severity in crude or multivariable-adjusted model in men and in the whole population.

**Table 3:** Ordinal logistic regression for the association between dietary acid-base indices and depression

Variable		PRAL				DAL			
		T1	T2	T3	p-trend	T1	T2	T3	p-trend
Female	Crude	1	1.03 (0.90–1.19)	1.13 (0.98–1.31)	0.090	1	**1.16 (1.01–1.33)**	**1.20 (1.03–1.39)**	**0.01**
	Model 1	1	1.02 (0.89–1.18)	**1.15 (1.00-1.33)**	**0.056**	1	**1.15 (1.00-1.132)**	**1.24 (1.07–1.44)**	**0.004**
	Model 2	1	1.00 (0.87–1.16)	1.22 (0.97–1.29)	0.127	1	1.13 (0.98–1.30)	**1.23 (1.05–1.43)**	**0.007**
	Model 3	1	1.04 (0.90–1.20)	**1.17 (1.00-1.36)**	**0.042**	1	1.15 (0.99–1.32)	**1.21 (1.03–1.42)**	**0.015**
Male	Crude	1	1.02 (0.85–1.22)	0.96 (0.80–1.14)	0.613	1	0.99 (0.97–1.02)	0.93 (0.77–1.12)	0.410
	Model 1	1	1.02 (0.85–1.23)	0.97 (0.81–1.16)	0.752	1	0.99 (0.81–1.21)	0.95 (0.77–1.14)	0.546
	Model 2	1	1.06 (0.88–1.27)	0.95 (0.79–1.14)	0.556	1	1.02 (0.83–1.24)	0.93 (0.77–1.27)	0.392
	Model 3	1	1.08 (0.89–1.30)	0.97 (0.80–1.17)	0.722	1	1.04 (0.85–1.27)	0.96 (0.79–1.05)	0.598
Total	Crude	1	1.00 (090-1.12)	1.01 (0.90–1.13)	0.839	1	1.05 (0.94–1.17)	0.95 (0.85–1.06)	0.351
	Model 1	1	0.99 (0.89–1.10)	1.05 (0.94–1.17)	0.408	1	1.04 (0.93–1.16)	0.99 (0.89–1.11)	0.920
	Model 2	1	**0.99 (1.13–1.15)**	1.02 (0.91–1.14)	0.712	1	1.05 (0.94–1.18)	1.02 (0.90–1.14)	0.756
	Model 3	1	1.03 (0.91–1.15)	1.07 (0.95–1.20)	0.274	1	**1.07 (1.05–1.20)**	1.00 (0.89–1.13)	0.896

Crude: unadjusted, Model1: adjusted for age and energy intake, Model2: Model1 + education level, smoking status, physical activity level, chronic diseases including diabetes, hypertension, or dyslipidemia, and marriage status, Model3: Model2 + BMI, fiber, MUFA. DAL: dietary acid load, PRAL: potential renal acid load

The multivariable-adjusted odds ratios (ORs) and 95% confidence interval (CIs) for anxiety severity across tertiles of DAL and PRAL are indicated in Table 4. Regarding the association between PRAL and anxiety severity, there was no significant association when separated by gender status. However, in the fully adjusted model for the whole population, participants in the highest tertiles of PRAL had 13% greater odds of more severe anxiety than the lowest tertiles (OR: 1.13, 95% CI: 1.01–1.13).

No significant association was observed between DAL and anxiety severity in crude or multivariable-adjusted model in women, men and in the whole population.

**Table 4:** Ordinal logistic regression for the association between dietary acid-base indices and anxiety

Variable		PRAL				DAL			
		T1	T2	T3	p-trend	T1	T2	T3	p-trend
Female	Crude	1	1.00 (0.87–1.15)	1.09 (0.95–1.26)	0.240	1	1.02 (0.86–1.17)	1.09 (0.94–1.26)	0.280
	Model 1	1	0.98 (0.85–1.28)	1.10 (0.95–1.27)	0.189	1	1.01 (0.88–1.16)	1.11 (0.96–1.29)	0.179
	Model 2	1	0.96 (0.83–1.10)	1.08 (0.93–1.24)	0.316	1	1.00 (0.88–1.16)	1.11 (0.95–1.29)	0.199
	Model 3	1	1.00 (0.87–1.17)	1.14 (0.98–1.32)	0.089	1	1.02 (0.88–1.26)	1.08 (0.93–1.26)	0.326
Male	Crude	1	1.21 (1.00-1.46)	1.20 (0.99–1.44)	**0.067**	1	1.15 (0.94–1.41)	1.11 (0.92–1.44)	0.342
	Model 1	1	1.20 (0.99–1.45)	1.18 (0.98–1.43)	**0.088**	1	1.14 (0.93–1.39)	1.02 (0.90–1.32)	0.466
	Model 2	1	1.18 (0.98 − 0.43)	1.15 (0.95–1.39)	0.169	1	1.11 (0.91 − 0.37)	1.04 (0.86–1.27)	0.766
	Model 3	1	1.20 (0.99–1.45)	1.17 (0.97–1.42)	0.121	1	1.13 (0.92–1.32)	1.08 (0.88–1.32)	0.546
Total	Crude	1	1.03 (0.92–1.15)	1.06 (0.95–1.18)	0.302	1	1.00 (0.89–1.11)	0.92 (0.82–1.02)	0.140
	Model 1	1	1.00 (0.90–1.13)	1.02 (0.98–1.22)	0.127	1	0.98 (0.88–1.09)	0.95 (0.85–1.90)	0.404
	Model 2	1	1.00 (0.90–1.12)	1.07 (0.95–1.20)	0.246	1	0.99 (0.89–1.11)	0.99 (0.88–1.10)	0.810
	Model 3	1	1.05 (0.93–1.17)	1.13 (1.01–1.13)	**0.037**	1	1.01 (0.90–1.13)	0.97 (0.86–1.09)	0.636

Crude: unadjusted, Model1: adjusted for age and energy intake, Model2: Model1 + education level, smoking status, physical activity level, chronic diseases including diabetes, hypertension, or dyslipidemia, and marriage status, Model3: Model2 + BMI, fiber, MUFA. DAL: dietary acid load, PRAL: potential renal acid load

## Discussion

According to our results, no significant differences were observed in the prevalence of depression and anxiety categories across tertiles of DAL. Women in the highest tertiles of PRAL and DAL had higher odds of depression than those in the lowest categories. Further, the subjects in the highest tertile of PRAL had higher anxiety risk than the lowest tertile in the fully-adjusted model (model 3). Also, we observed that higher consumption of fruits, vegetables, and fiber and a lower intake of meats and grains was associated with lower DAL score. These findings are in line with some previously published studies [14, 39]. There are limited studies evaluating the relationship between diet and depression with attention to the DAL.

In contrast with our findings, in a cross-sectional study conducted on 4378 adults, participants in the highest DAL group had 100% and 92% higher risk of depression and anxiety than the lowest DAL group, respectively, and this association remained significant after adjustment for confounding factors [23]. Another cross-sectional study on 447 Iranian females has shown a strong positive relationship between dietary acid-base load and depression, anxiety, and psychological distress. Similar to our results, women who had higher scores of DAL and PRAL had higher odds of depression compared to the lower scores [22].

Numerous scientific studies have established a strong connection between diet and mental health [40–43]. A growing body of evidence indicates that a poor diet characterized by the consumption of processed foods, refined sugars, and unhealthy fats can have detrimental effects on mental well-being [44, 45]. Such a diet has been linked to an increased risk of developing and exacerbating mental health disorders, including depression and anxiety [46, 47]. Conversely, a healthy and balanced diet, encompassing a variety of fruits, vegetables, lean proteins, and essential nutrients like omega-3 fatty acids, vitamins, and minerals, has been associated with improved mental health outcomes [48, 49].

There is limited scientific evidence specifically linking acidic foods to mood regulation [20, 22, 23]. However, some studies suggest that the overall acidity or alkalinity of the diet, as measured by the PRAL, may have an impact on mental health [50]. A high PRAL diet, which is typically characterized by the consumption of animal proteins, processed foods, and refined sugars, has been associated with increased inflammation in the body [51, 52]. Chronic inflammation has been linked to a higher risk of developing mental health disorders, including depression and anxiety [53]. On the other hand, a low PRAL diet, which includes fruits and vegetables, has been associated with reduced inflammation and improved mental health outcomes [54, 55]. While the specific role of acidic foods in mood regulation is not well-established, it is important to maintain a balanced diet that includes a variety of nutrient-rich foods to support optimal mental health. Further research is needed to better understand the relationship between dietary acidity and mood regulation.

Also, there is growing evidence to suggest that diet can influence the activity and expression of acid-sensing ion channels (ASICs) [56, 57]. The ASIC is a class of ion channels that are sensitive to changes in extracellular acidity. These channels play a crucial role in various physiological processes, including sensory perception, neuronal excitability, and synaptic transmission [58]. Certain dietary factors, such as high intake of acidic foods or low intake of alkaline foods, can alter the pH balance in the body and potentially affect ASIC function [59, 60]. A diet high in acidic foods, such as processed meats, may lead to increased extracellular acidity and subsequently activate ASICs. On the other hand, a diet rich in alkaline foods, such as fruits and vegetables, may promote a more alkaline environment and potentially modulate ASIC activity [61, 62]. Additionally, certain nutrients and dietary components, such as omega-3 fatty acids and antioxidants, have been shown to influence ASIC expression and function [63].

Emerging evidence suggests that ASICs may also be involved in the pathophysiology of depression [64, 65]. Studies have shown that ASIC expression and activity are altered in animal models of depression, as well as in postmortem brain samples from individuals with depressive disorders [66, 67]. Furthermore, pharmacological modulation of ASIC activity has been found to influence depressive-like behaviors in animal models. It is believed that ASICs may contribute to the regulation of mood through their involvement in neurotransmitter release, neuronal plasticity, and neuroinflammation [68, 69]. However, the exact mechanisms by which ASICs influence depression are not yet fully understood and require further investigation. Nonetheless, these findings highlight the potential importance of acid sensing ion channels in the development and treatment of depression. Further research is needed to fully understand the complex interplay between diet and ASICs and its implications for health and disease, including depression. However, these findings suggest that dietary interventions targeting ASICs may hold promise for the prevention and treatment of various disorders, including depression.

The main strength of the current study was using validated questionnaires to evaluate the participants’ dietary intake and mental health. Further, the large sample size of the study was another strength. It can be noted that most of the recent studies were focused on only one index to evaluate the acid load of the diets, while in the current study, two indices (DAL and PRAL) were used. Also, the current study has some limitations that must be noted. Our investigation was located in a specific area, so the results cannot be generalized to other regions. In addition, mental illnesses such as depression are affected by several other factors that have not been assessed in this study. For example, it would be better to assess the economic status of the participants, which is an influential factor in mental health. Further, the present study’s cross-sectional nature prevented us from drawing any causal linkage. Furthermore, the assessment of dietary intake and mental health by questionnaires may have overstatement or understatement that increases the rate of misclassification in the study.

## Conclusion

We found that women, but not men, with higher dietary acid load had significantly higher odds of having more severe depression. A significant positive association was also observed between dietary acid load and anxiety in the whole population. To confirm these findings, it is necessary to conduct studies of a prospective nature. Future studies should compare the effects of diet-based acid load on mental health, considering different dietary patterns.

## Data Availability

The datasets generated and/or analyzed during the current study are not publicly available due to university data ownership policies, but are available from the corresponding author on reasonable request.
